# Bilateral Blunt Traumatic Dissections of the Extracranial Internal Carotid Artery: A Case Report and Literature Review

**DOI:** 10.7759/cureus.53630

**Published:** 2024-02-05

**Authors:** Yahya H Khormi, Atheer I Darraj, Alshaymaa Arishy, Seham O Abuzahirah, Mostafa Atteya

**Affiliations:** 1 Neurological Surgery, Jazan University, Jazan, SAU; 2 Internal Medicine, King Fahad Central Hospital, Jazan, SAU; 3 Medicine and Life Sciences, Maastricht University, Netherlands, NLD; 4 Neurosurgery, King Fahad Hospital, Jeddah, SAU; 5 Neurological Surgery, King Fahad General Hospital, Jazan, SAU

**Keywords:** dissections, vascular dissection, traumatic brain injury, internal carotid artery, cervical

## Abstract

Bilateral traumatic dissections of the cervical internal carotid artery (ICA) are rare complications of polytrauma. A thorough literature review was performed, and data from selected studies were analyzed to assess the trends in clinical presentation, modes of trauma, management protocols, and clinical outcomes. The reported outcomes were categorized and graded into optimal, intermediate, and poor outcomes. We describe a rare case of bilateral dissection of ICA in a 31-year-old woman who was involved in a motor vehicle accident. She had a Glasgow Coma Scale score of 9 and right-sided hemiparesis. Radiological findings revealed left upper ICA dissection, arterial intramural thrombus, and stenosis of the upper segment of the right ICA. She improved on conservative management and had a good clinical outcome at a three-month follow-up. Emergency physicians must be knowledgeable about such cases, as more than half of these trauma victims are initially asymptomatic on initial presentation. Specific diagnostic and therapeutic modalities should be implemented based on low threshold clinical suspicion to avoid missing these potentially disabling injuries and reduce morbidity and mortality. Computed tomographic angiography is recommended in cases with atypical clinical presentations, unexplained neurological deficits, or delayed-onset clinical deterioration. While antiplatelet and anticoagulant therapies are the mainstays of conservative management, endovascular and surgical management are only used in severe cases when medical treatment has failed, the artery has been completely transected, or there is active bleeding. Generally, good outcomes were reported in about two-thirds of those patients.

## Introduction

Carotid artery dissection is a relatively rare condition, as the prevalence is between 2.5 and three per 100,000/year [1]. The segment of the internal carotid artery (ICA), which is located between the bifurcation of the common carotid artery and the base of the skull, is referred to as the cervical ICA [2]. Even though the ICA's location places it at a significant risk for injury due to its mobility and susceptibility to stretching, the majority of carotid artery dissections occur spontaneously, and only 4% of the reported dissections of the ICA are related to severe trauma [3,4]. The most common type of trauma is blunt trauma, which is typically caused by accidents involving motor vehicles and can disrupt one or more of the layers of the ICA [5,6]. Research studies have demonstrated that almost 1% to 2% of patients who suffered blunt trauma had extracranial traumatic vascular injuries, while the incidence of ICA injuries was between 0.08% and 0.33%. However, fortunately, 52%-79% of these injuries did not manifest any symptoms [7,8].

In most patients with ICA dissection, conservative medical treatment is sufficient for effective management [9]. However, on the other hand, endovascular and surgical interventions are required in a relatively small percentage of cases. The diagnosis of traumatic dissection of the ICA is frequently challenging and may be delayed because the presenting symptoms may be overlooked in the initial clinical assessment, particularly in the presence of other injuries [3]. Blunt bilateral traumatic dissections of the ICA are a very uncommon entity, as evidenced by the limited number of published case reports and series in the medical literature. However, highlighting such injuries is essential to improving the awareness of clinicians and the involved healthcare team, as it can effectively aid in increasing the suspicion index for diagnostic and treatment strategies, which in turn can reduce the associated morbidity and mortality of trauma patients. We report a rare case of bilateral dissection of ICA in a 31-year-old woman. Additionally, we compare the findings of our reported case with those of the 40 patients who were reported in 26 different papers in the literature to signify the importance of such injuries in terms of presentation, diagnosis, and management [10-36]. These studies from the literature were retrieved after conducting an intensive review and analysis of the available literature.

## Case presentation

**Figure 1:**
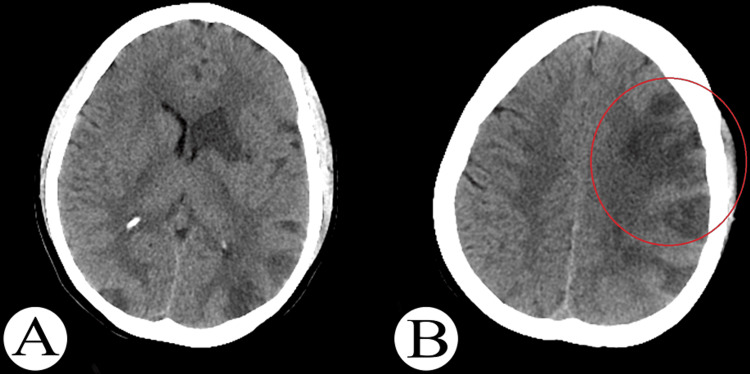
(A, B) Initial post-traumatic axial computed tomography of the brain showing multiple bilateral hemispheric hypodense area multiple strokes (red circle).

**Figure 2:**
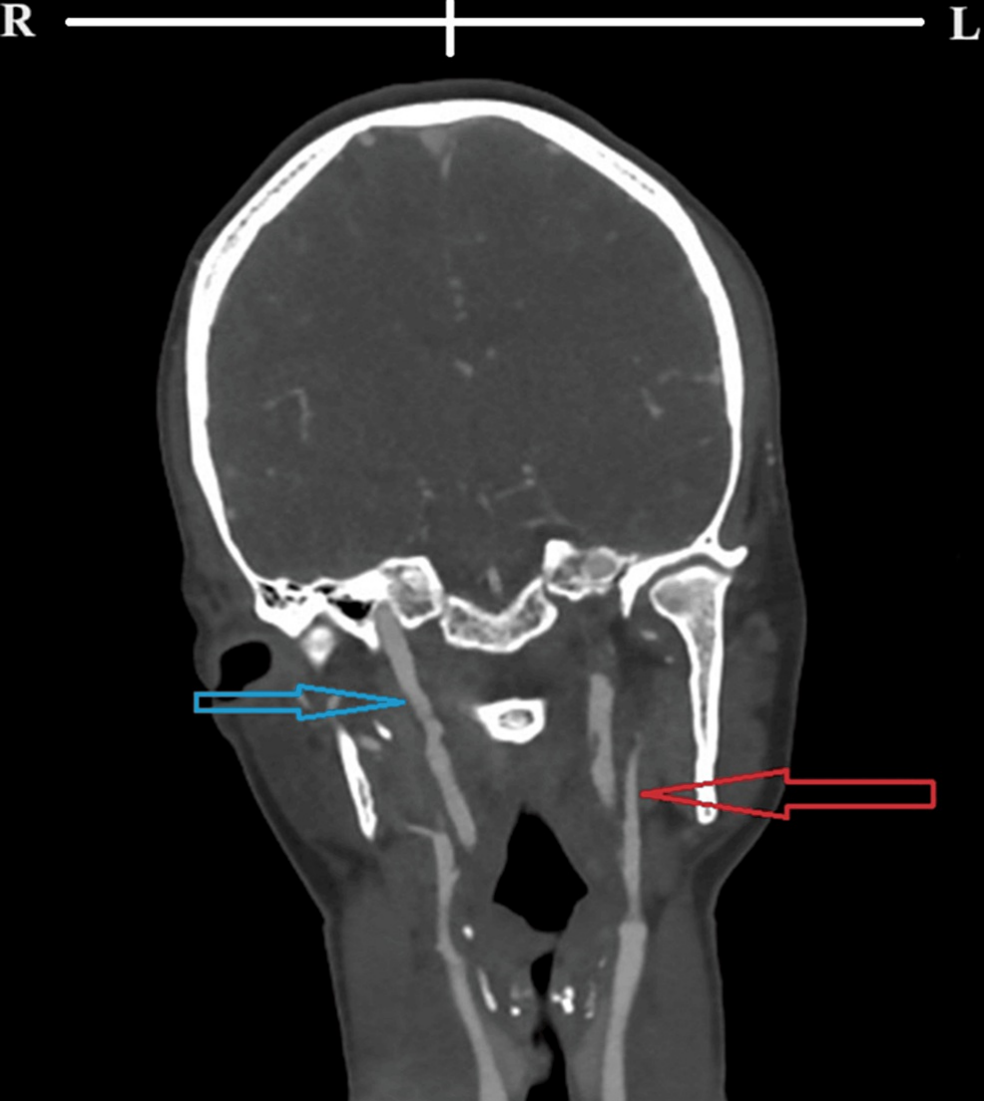
Initial coronal computed tomography angiogram of the neck showing left upper cervical ICA dissection (red arrow) and evidence of arterial intramural thrombus, in addition to stenosis of the upper segment of right ICA (blue arrow) R: right side; L: left side; ICA: internal carotid artery

A 31-year-old woman was involved in a motor vehicle collision and was referred to our facility by a nearby hospital 24 hours after the incident. The patient's medical history was unremarkable, and she did not take any regular medications. On arrival, her vital signs were stable, and she had a Glasgow Coma Scale (GCS) score of 9/15. She was localizing to pain, opening her eyes in response to speech, and demonstrating no verbal response. She had grade 3 right-sided hemiparesis on the Medical Research Council scale for motor power. Cranial nerves were grossly intact. In addition, distal arterial pulsations were normal, and there were no signs of arterial ischemia in any of the extremities. Due to the patient's atypical presentation and unexplained neurological deficits, we proceeded from plain brain computed tomography (CT) to CT angiography, magnetic resonance imaging, and magnetic resonance arteriography. The images revealed left upper ICA dissection, arterial intramural thrombus, and stenosis of the upper segment of the right ICA. The skeletal examination revealed stable sacral and pubic rami fractures, and the abdomen CT scan revealed a low-grade liver injury (Figures 1A, 1B, 2).

**Figure 3:**
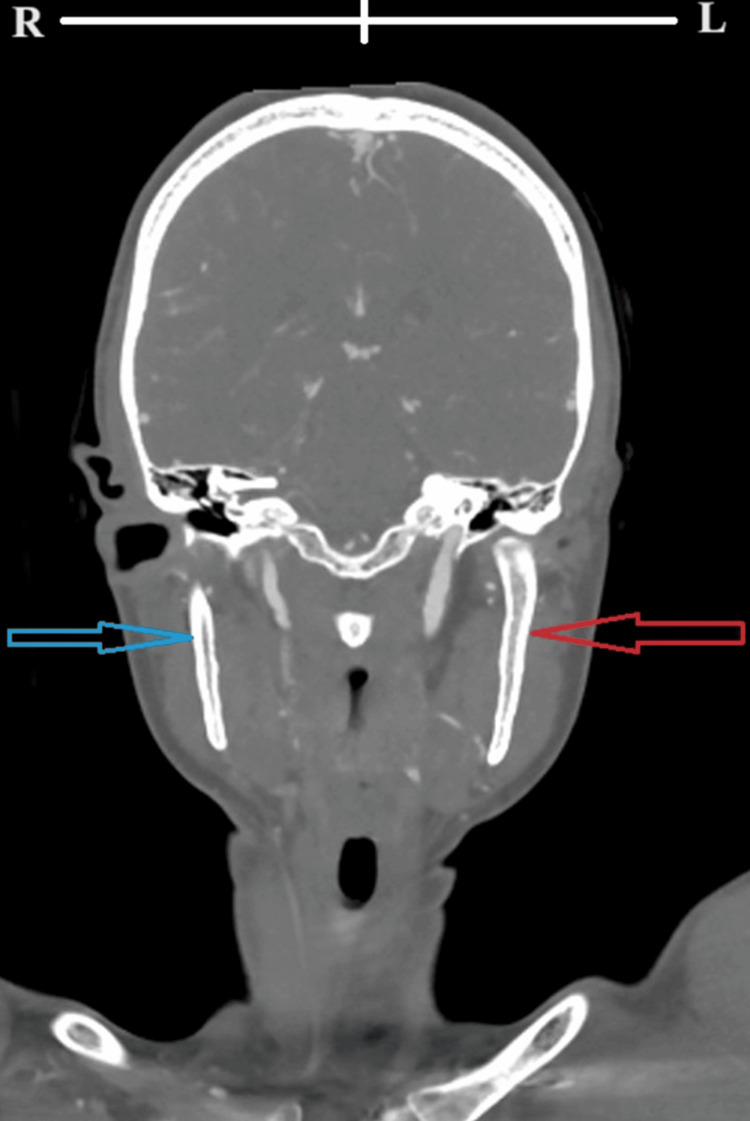
A follow-up coronal computed tomography angiogram of the neck showing normal left internal carotid artery (red arrow) and mild residual right internal carotid stenosis (blue arrow). R: right; L: left

Antiplatelet therapy with aspirin 81 mg once daily and therapeutic subcutaneous doses of enoxaparin 6,000 IU twice daily for three months was initiated. Liver injury and fractures of the sacral and pubic rami were treated conservatively without complications. The patient's condition gradually improved under conservative care until she became fully awake, conscious, and verbally responsive. Both aphasia and right-sided hemiparesis improved gradually. She was discharged two weeks following admission. Before discharge, follow-up CT angiography revealed the disappearance of filling defects in the left internal artery and the persistence of stenosis in the upper right ICA (Figure 3). On a three-month follow-up, the patient showed no residual neurological deficits.

## Discussion

The majority of ICA dissections are spontaneous [3]; however, there is limited clinical data on ICA dissections caused by trauma. Moreover, the bilateral occurrence of traumatic ICA dissections is exceedingly uncommon, and our current case is therefore regarded as an important addition to the current literature on this rare vascular disorder [37]. Our comprehensive review yielded information including clinical presentation, diagnosis, management strategies, and clinical outcomes.

While studying age and gender distribution among our study cases, we identified that four patients were under the age of 18 at the time of injury, 29 patients were between the ages of 18 and 40, and seven patients were older than 40 years. The average age of the patients studied was 31 years. Twenty-three of the patients in the study were female. Cranial nerve palsies, alone or in combination, were observed in 12 patients. The cranial nerve palsies most frequently reported were facial nerve palsy (nine patients), oculomotor nerve palsy (two patients), and glossopharyngeal nerve palsy (one patient).

Motor vehicle accidents were the leading cause of injury in almost 80% of 32 cases. Other causes reported of trauma were domestic violence (7.5%) and one case each of horse kicks, falling from a height, neck strangulation injury, chiropractic manipulation, and a private aircraft accident. Cronlein et al. reported that the sudden deceleration of motor vehicles is likely the cause of traumatic ICA dissections in restrained passengers [21]. While activation of the coagulation cascade, cerebral arterial dissection increases the risk of developing a thrombus as well as cerebral thromboembolism and ischemia [3,38,39]. The clinical presentation of observed cases was marked by substantial variation and discordance from being asymptomatic, having early neurological deficits, or having a clinical presentation with a delayed onset.

The group of asymptomatic patients, however, is quite challenging and is frequently identified by accident or overlooked. Patients typically exhibit no neurological deficits. Findings from our review of the literature revealed that almost 55% (22 patients) were initially asymptomatic, and among them, 17 were aged between 18 and 40 years. In seven asymptomatic patients, symptoms started between six and 12 hours; in nine patients, symptoms started between one and seven days; and in five patients, symptoms started one to six months following trauma. One patient had a delayed onset of symptoms, but the duration was not specified.

In the group of patients who presented with early symptoms, there were 18 patients (45%) with immediate post-traumatic symptoms or impairments. Hemiparesis (n = 15), pupillary changes, visual field cuts, cranial nerve palsies (n = 15), aphasia (n = 10), and coma (n = 4) were among the presenting symptoms. Headache, dizziness, neck pain, amaurosis fugax, pronator drift, hemi-hypoesthesia, Horner's syndrome, and incoordination were also reported as less frequent manifestations. The initial and delayed presentation of cases is demonstrated in Table 1.

**Table 1:** Initial and delayed presentation of cases

Author and year	Case No.	Initial clinical presentation	Delayed clinical presentation	Time between initial and delayed presentation
Malek AM, 2000 [10]	1	Asymptomatic	Rt hand & arm paresis, Dysphasia	3months
	2	Asymptomatic	Lt hand & arm paresis, Pronator drift	3months
	3	Asymptomatic	Coma, Bilateral sensorimotor dysfunction, Aphasia	6 months- 1 year
Chang C, 2017 [11]	4	Asymptomatic	Hemiparesis, and Aphasia	1week
Agarwal A, 2020 [12]	5	Asymptomatic	Lt sided weakness	2week
Taoussi N, 2017 [13]	6	Asymptomatic	Motor dysphasia, Rt upper limb paresis	8hours
Ballard JL, 1992 [14]	7	Asymptomatic	Rt (Facial palsy, Hemiparesis) and expressive aphasia	Few hours
Gabriel SA, 2019 [15]	8	Severe headache, Dizziness, Cervical pain, and Rt amaurosis fugax	Lt Hemiplegia, Aphasia, Dysphagia and Rt facial palsy	48hours
Bajkó Z, 2016 [16]	9	Asymptomatic	hemiplegia, Hemi hypoesthesia	12hours
Gioia S, 2019 [17]	10	Asymptomatic	Restless, Disorientation and aphasia	1week
Thie A, 1993 [18]	11	Asymptomatic	Aphasia, Rt hemiparesis, Lt Horner’s syndrome	7days
	12	Asymptomatic	Left hemiparesis	2days
	13	Coma, Midbrain syndrome	never recovered	4hours
	14	Asymptomatic	Upper limb weakness	.
	15	Asymptomatic	Lt hemiparesis	7hours
	16	Coma	Rt hemiparesis	2days
	17	Asymptomatic	9th nerve palsy	1months
	18	Coma, Midbrain syndrome	Rt hemiparesis	1months
	19	Asymptomatic	Upper limb weakness	6days
	20	Coma	Lt hemiparesis	12hours
	21	Asymptomatic	Iatrogenic coma with minor lateralizing signs	2days
Fanelli F, 2004 [19]	22	Hemiplegia	.	.
Chomel A, 2002 [20]	23	Asymptomatic	Lt hemiplegia	1day
	24	Asymptomatic	Drowsiness, Rt hemiparesis, Clonic convulsion of Rt upper limb, and Rt Horner syndrome	6days
Crönlein M, 2015 [21]	25	Severe headache	.	.
Duncan MA, 2000 [22]	26	Homonymous hemianopia, Hemiplegia and facial palsy	.	.
Petetta C, 2019 [23]	27	Traumatic shock condition, vital signs: pulse: 120 beats/min; blood pressure:60/40 mm Hg; and SpO2, 70%.	Mixed aphasia	1week
Pittock SJ, 2001 [24]	28	facial palsy, Hemiparesis, Hemineglect syndrome	.	.
Yong RL and Heran NS, 2005 [25]	29	Dilated pupil	.	48hours
Watridge CB, 1989 [26]	30	Aphasia and hemiparesis	.	.
Stringer LW, 1980 [27]	31	Asymptomatic	Confusion, Aphasia	8hours
Storrow AB and Smith BA, 1995 [28]	32	Asymptomatic	Rt hemiparesis, Expressive aphasia	12hours
Scherman BM, 1982 [29]	33	Asymptomatic	Hemiparesis	6hours
Malin J-P, 1985 [30]	34	Asymptomatic	Rt hemiplegia and facial nerve paresis, Aphasia, Agraphia and dyslexia	5months
Molacek J, 2010 [31]	35	Dilated pupils	Tonic-clonic seizure	13days
Nadgir RN, 2003 [32]	36	Rt ptosis, Lt Facial palsy and paresthesia	Loss of coordination, hypoesthesia and involuntary movement of U and L limb and dysarthria	.
Robinson RG and Gwynne JF, 1978 [33]	37	Unconscious with right-sided weakness, Partial left ptosis	Deterioration of symptoms	4days
de Borst GJ, 2006 [34]	38	Unconscious	Facial palsy, paralysis, hemianopia	Few days later
Friedenberg MJ, 1973 [35]	39	Hypotensive, Agitated, and confused with constricted pupils	.	.
Fukuda I, 1989 [36]	40	Shock, and cyanotic extremities	.	.

There are numerous diagnostic imaging modalities for assessing vessel dissection [21]. CT scans and CT angiography are primarily used to diagnose cerebral vascular pathologies in emergencies. String signs, lupus constrictions, and arterial hypertension are regarded as indirect indicators of an artery dissection [38]. In the reported cases, various diagnostic techniques were utilized to confirm the diagnosis. In 17 cases, carotid angiography was the most frequently used imaging modality, followed by CT angiography in 15 cases (Table 2).

**Table 2:** Neurovascular imaging findings in the reviewed articles DSA: Digital Subtraction Angiography; CT: Computed tomography; MRA: Magnetic resonance angiography; ICA: Internal carotid artery

Author and year	Case No.	Brain images findings	Vascular image types- findings
Malek AM, 2000 [10]	1	Hemispheric embolic infarction	Digital Subtraction Angiography (DSA)-Bilateral symmetrical focal stenoses in ICA at C2-C3 level
	2	Opercular infarction	Digital Subtraction Angiography (DSA)-Bilateral symmetrical focal stenosis in ICA at C2-3 level
	3	Bilateral frontal infarctions watershed distribution	Digital Subtraction Angiography (DSA)-Lt: extracranial ICA occlusion; Rt: extracranial ICA chronic dissection
Chang C, 2017 [11]	4	Sub insular cortex and lentiform nucleus infarction	MRA-Bilateral extracranial internal carotid artery dissections with aneurysms formation
Agarwal A, 2020 [12]	5	caudate nucleus, Insula, and parietal lobes infarction	CTA-Dissection of bilateral distal cervical ICA
Taoussi N, 2017 [13]	6	Frontal lobe infarction	CTA-Bilateral occlusion of the ICA
Ballard JL, 1992 [14]	7	Posterior fronto-parietal infarction	Carotid Angiography-Bilateral ICA dissections above bifurcations and extending to skull base
Gabriel SA, 2019 [15]	8	corona radiata infarction	Carotid Angiography-String sign in the distal Rt and Lt internal carotid arteries
Bajkó Z, 2016 [16]	9	MCA infarction	Ultrasound examination of the carotid arteries-Rt ICA: a high-resistance flow signal, without stenotic lesions at the proximal level, suggestive of significant distal stenosis or occlusion, Lt ICA: irregular stenosis caused by a hypoechoic mural thickening, suggestive of a mural hematoma secondary to dissection
Gioia S, 2019 [17]	10	Cortical-subcortical infarction	CTA-Bilateral dissection of the extracranial internal carotid arteries
Thie A, 1993 [18]	11	.	Angiography-Bilateral carotid artery stenosis
	12	.	Angiography-Bilateral aneurysm at base of the skull
	13	.	Angiography-Bilateral stenosis of extracranial internal carotid
	14	.	Angiography-Rt stenosis at C1/C2, Lt not done; filling of left anterior cerebral artery from right ICA raised suspicion of left ICA lesion
	15	.	Angiography-Rt stenosis at C2, Lt occlusion at C2.
	16	.	Angiography-Rt stenosis at the base of the skull, Lt aneurysm
	17	.	Angiography-Bilateral carotid artery stenosis
	18	.	Angiography-Bilateral carotid artery stenosis
	19	.	Angiography-Bilateral carotid artery stenosis
	20	.	Angiography- Rt: Occlusion at base of the skull, Lt: stenosis and two aneurysms at base of the skull
	21	.	Angiography-Rt: tapering occlusion at C2, Lt: occlusion and aneurysm at base of the skull
Fanelli F, 2004 [19]	22	Hemispheric infarction	DSA-Rt: dissection and obstruction of the right ICA with its reconstitution at the level of the intracranial tract, Lt: pseudoaneurysm associated with the dissection of the left ICA.
Chomel A, 2002 [20]	23	Frontal, Peduncular and capsular infarction.	CTA-Rt internal carotid dissection up to the entrance in the carotid channel, and Lt internal carotid dissection with pseudoaneurysm
	24	Caudate nucleus infarction	CTA-Lt: dissection of the left ICA with extension to the intracranial segment, Rt: dissection of the right ICA just below the petrosal bone with false aneurysm.
Crönlein M, 2015 [21]	25	Bi-hemispheric, mainly left-sided infarction	CTA-Bilateral internal carotid artery dissection
Duncan MA, 2000 [22]	26	parietal lobe infarction	CTA-bilateral ICA dissection, fibromuscular dysplasia of the ICAs and thrombus in Rt ICA
Petetta C, 2019 [23]	27	Multiple extensive ischemic areas in the frontal region and circumscribed lesions in the parietal region bilaterally, in the left occipital region, and in the left thalamic site.	CTA-Bilateral internal carotid artery dissection
Pittock SJ, 2001 [24]	28	Anterior cerebral artery territory infarction	Digital Subtraction Angiography (DSA)-Bilateral internal carotid artery dissection
Yong RL and Heran NS، 2005 [25]	29	Bi-cerebellar hemispheres infarction, Rt parietal, and Lt frontal subcortical white matter infarction.	CTA-Bilateral internal carotid artery dissection
Watridge CB, 1989 [26]	30	Normal	CTA-Bilateral internal carotid artery dissection
Stringer LW, 1980 [27]	31	Normal	CTA-Bilateral internal carotid artery dissection
Storrow AB and Smith BA, 1995 [28]	32	Parietal region infarction	CTA-Bilateral internal carotid artery dissection
Scherman BM, 1982 [29]	33	.	Carotid Arteriography-Bilateral internal carotid artery dissection
Malin J-P, 1985 [30]	34	Hemispheric infarction	Carotid angiography-Bilateral internal carotid artery dissection
Molacek J, 2010 [31]	35	.	CTA-Bilateral internal carotid artery dissection
Nadgir RN, 2003 [32]	36	Thalamic infarction	MRA-Bilateral internal carotid artery dissection
Robinson RG and Gwynne JF, 1978 [33]	37	Parietal and occipital areas, Internal capsule, and basal ganglia infarction	Arteriogram-Bilateral ICAs were thrombosed from about 2 cm above the common carotid artery bifurcations.
de Borst GJ, 2006 [34]	38	Cerebral hemisphere and anterior cerebral artery infarction	CTA-Bilateral stenosis of extracranial internal carotid artery
Friedenberg MJ, 1973 [35]	39	.	Angiogram-Bilateral internal carotid arteries occlusion
Fukuda I, 1989 [36]	40	Frontal and occipital lobe infarction	CTA and DSA-Bilateral internal carotid artery dissection

Anticoagulants and antiplatelet medications were used to treat most patients [3,40], eight underwent endovascular procedures, and three underwent surgery after medical treatment failed (Tables 3, 4). Cerebrovascular dissections can be treated with open surgical methods like microvascular suturing, extracranial-intracranial bypass, and thromboendarterectomy, or endovascular methods like stenting, stent-assisted intravascular thrombolysis, and thrombectomy [3,38]. Surgical and endovascular alternatives are used in the most severe and critical clinical situations [40], in cases with complete arterial transactions, and after medical treatment fails [41].

**Table 3:** Summary of the medical and surgical interventions of 40 patients in the reviewed articles

Type of management		Specified management	Frequency	Percent
Medical management	Antiplatelets / Anticoagulant		29	72.5
	Not mentioned		11	27.5
Surgical interventions	Endovascular intervention		8	20
	Emergency operation		3	7.5

**Table 4:** Management details and clinical outcomes of 40 patients in the reviewed articles

Author and year	Case No.	Management (Medical)	Management (Intervention)	Time of last follow up	Radiological outcome	Clinical outcome
Malek AM, 2000 [10]	1	Clopidogrel (75 mg daily) or ticlopidine hydrochloride-before the procedure, and daily aspirin.	Angioplasty and stent placement	8months	Persistent patency of the stented Lt ICA with no evidence of intimal hyperplasia or mismatch with the native artery	Sustained retroperitoneal hemorrhage
	2	Clopidogrel (75 mg daily) or ticlopidine hydrochloride-before the procedure, and daily aspirin.	Percutaneous Balloon Angioplasty	20months	.	Asymptomatic, no further neurological deficits.
	3	Anticoagulation therapy consisting of heparin, later changed to warfarin.	.	3monhs	.	Persistent paralyzed right upper extremity and weak left U & L extremity, mild dysphasia.
Chang C, 2017 [11]	4	Aspirin initially	Carotid Wallstent endoprostheses	1week	.	Recovered
Agarwal A, 2020 [12]	5	Antiplatelets (aspirin, clopidogrel) and physiotherapy	.	2week	.	Improved MRC 3/5
Taoussi N, 2017 [13]	6	Clexane and LMWH, warfarin after 7 days and continued for 6 months	.	.	.	Improvement with minor dysphasia
Ballard JL, 1992 [14]	7	Heparin	Rt side revascularization, Autologous right, ICA bypass	15months	Widely patent Rt graft and return the intracerebral flow to normal	Recovered
Gabriel SA, 2019 [15]	8	Antiplatelets (aspirin) , therapeutic intravenous heparin followed by warfarin for 6 months	.	.	.	Improvement with minor dysphasia and dyslalia
Bajkó Z, 2016 [16]	9	Aspirin and LMWH as prophylactic doses (initially, and 3 weeks following emergency decompressive craniotomy due to ischemic event)	.	.	.	Improved
Gioia S, 2019 [17]	10	Heparin then followed by warfarin	.	.	.	Recovered
Thie A, 1993 [18]	11	2 Patients received anticoagulant drugs during the acute phase	.	.	.	hemiparesis
	12	.	.	.	.	hemiparesis
	13	.	.	.	.	Death
	14	.	.	.	.	No deficits
	15	.	.	.	.	Death
	16	.	.	.	.	Death
	17	.	.	.	.	No deficits
	18	.	.	.	.	hemiparesis
	19	.	.	.	.	No deficits
	20	.	.	.	.	hemiparesis
	21	.	.	.	.	Slight cognitive impairment
Fanelli F, 2004 [19]	22	Heparin before procedure, discharge on ticlopidine and LMWH	Carotid Wallstent endoprostheses	13months	Good recanalization of the right ICA	Mild Lt arm weakness
Chomel A, 2002 [20]	23	Anticoagulant: 2 weeks of heparin, followed by LMWH as prophylactic use. Ten days later, antiplatelet drugs were introduced.	.	1month	Persistent, localized stenosis of the right internal carotid with a false aneurysm. The left internal carotid was normal.	hemiplegia
	24	Anticoagulant: 2 weeks of heparin, followed by LMWH (Therapeutic)+ 10 days of preventive use of LMWH, and long-term antiplatelet	.	.	Normalization of the left internal carotid artery	Recovered
Crönlein M, 2015 [21]	25	Heparin	Angioplasty	6months	Improved	Improved
Duncan MA, 2000 [22]	26	Aspirin, then heparin which changed to warfarin	.	.	.	faciobrachial palsy
Petetta C, 2019 [23]	27	Therapeutic enoxaparin	.	1year	.	hemiparesis
Pittock SJ, 2001 [24]	28	IV heparin, Oral warfarin	.	1year	.	Improved
Yong RL and Heran NS, 2005 [25]	29	Heparin infusion followed by warfarin	.	1year	Improved	Recovered
Watridge CB, 1989 [26]	30	Heparin	.	6months	Recanalization of the dissected left internal carotid artery and near-normal healing of the right internal carotid artery.	Mildly hand weakness and mild expressive aphasia
Stringer LW, 1980 [27]	31	Heparin started and replaced gradually with warfarin	.	<1year	.	Recovered
Storrow AB and Smith BA, 1995 [28]	32	Warfarin	.	.	Slight improvement in both internal carotid narrowing.	Recovered
Scherman BM, 1982 [29]	33	Heparin	Endovascular (Fogarty catheter)	.	.	Death
Malin J-P, 1985 [30]	34	.	.	5months	Improved	Recovered
Molacek J, 2010 [31]	35	.	Stents insertion into both ICAs	22days	Improved	Recovered
Nadgir RN, 2003 [32]	36	Heparin, discharged coumadin and aspirin.	.	Few weeks later	Improved	Recovered
Robinson RG and Gwynne JF, 1978 [33]	37	.	Rt mid-temporal burr hole was made under local anesthesia, there was no hematoma, the brain was not under tension, and when the left temporal horn of the ventricle was tapped, the fluid was clear and at low pressure.	.	No difference	Death
de Borst GJ, 2006 [34]	38	LMWH for 1 week, followed by antiplatelet therapy (carbasalate calcium)	.	6months	Improved	Recovered
Friedenberg MJ, 1973 [35]	39	Heparin, Digitalis, followed by coumadin	.	53days	Improved	Recovered
Fukuda I, 1989 [36]	40	Heparin	Emergency operation including vessels occlusion and ligation	6months	Improved	Recovered

We reviewed the available literature and classified the reported clinical outcomes as good outcomes when there had been complete recovery or only mild neurological deficits, intermediate outcomes when there was hemiparesis, and poor outcomes when there were severe permanent deficits or death. Overall, among all 40 patients assessed, optimal, intermediate, and poor outcomes were observed in 25, eight, and seven patients, respectively, and in total, seven mortalities were noted (Table 4). Among 22 initially asymptomatic patients, optimal, intermediate, and poor outcomes were reported in 14, five, and three patients, respectively. In this group, one mortality was observed. On the other hand, among 18 initially symptomatic patients, optimal, intermediate, and poor outcomes were encountered in 11, three and four patients, respectively, and in this cohort, four mortalities were reported. In our case, we observed optimal clinical and radiological outcomes.

## Conclusions

Bilateral traumatic dissections of the extracranial cervical ICA occur in polytraumatized patients, and more than half of these cases are initially asymptomatic on initial presentation. Specific diagnostic and therapeutic modalities should be implemented based on low threshold clinical suspicion in order to avoid missing these potentially disabling injuries and reduce morbidity and mortality. CT angiography is recommended in cases with atypical clinical presentations, unexplained neurological deficits, or delayed-onset clinical deterioration. Antiplatelet and anticoagulant therapies are the mainstays of conservative management. Endovascular and surgical management are only used in severe cases when medical treatment has failed, the artery has been completely transected, or there is active bleeding. Generally, optimal outcomes were reported in about two-thirds of those patients. A multidisciplinary management approach is mandatory in this rare type of injury.
